# Supplementary material for: Persistence of Rare Salmonella Typhi Genotypes Susceptible to First-Line Antibiotics in the Remote Islands of Samoa
Source: mBio. 2022 Sep 12;13(5):e01920-22. doi: 10.1128/mbio.01920-22 (PMC9600463; doi:10.1128/mbio.01920-22)
Supplement: TEXT S1 [file mbio.01920-22-s0007.docx]

**Draft genome assembly**

**#Filter out Phi X 174 (E. coli bacteriophage)**

bbduk.sh in1=reads_1.fastq in2=reads_2.fastq out1=clean_1.fastq out2=clean_2.fastq ref= phix174_ill.ref.fa.gz k=31 hdist=1 stats=stats.txt

**#Trim and crop Illumina fastq data and remove adapters**

java -jar trimmomatic-0.38.jar PE -phred33 clean_1.fastq clean_2.fastq reads_1_paired.fastq reads_1_unpaired.fastq reads_2_paired.fastq reads_2_unpaired.fastq ILLUMINACLIP: illumina.adapters.current.fasta.txt:2:30:10 LEADING:3 TRAILING:3 SLIDINGWINDOW:4:15 MINLEN:36

**#Run SPAdes v3.14.1 with default settings**

/usr/local/packages/spades/bin/spades.py --isolate --pe1-1 reads_1_paired.fastq --pe1-2 reads_2_paired.fastq -o $f/$newdir

**Variant calling and core genome alignment**

**#Run Snippy v4.6.0 to call variants using hard thresholds against the Samoa reference**

snippy --cpus 16 --mincov 10 --minfrac 0.9 --outdir output.dir --ref LT904890.1.gbk --R1 *1.fastq.gz --R2 *2.fastq.gz

**#Run Snippy core to generate core genome alignment while masking for phage regions**

snippy-core --ref LT904890.1.gbk --prefix core --mask LT904890.1_phage_regions.bed snps1 snps2 snp3 snp4

**#LT904890.1_phage_regions.bed (available at https://phaster.ca/submissions/LT904890.1)**

LT904890 2061396 2067556

LT904890 2275822 2309188

LT904890 2795885 2840157

LT904890 3956048 3974131

LT904890 4149773 4198187

LT904890 4480780 4531589

**#Run Snippy v4.6.0 to call variants using hard thresholds against the CT18 reference**

snippy --cpus 16 --mincov 10 --minfrac 0.9 --outdir output.dir --ref AL513382.1.gbk --R1 *1.fastq.gz --R2 *2.fastq.gz

**#Run Snippy core to generate core genome alignment while masking for phage regions**

snippy-core --ref AL513382.1.gbk --prefix core --mask AL513382.1_phage_regions.bed snps1 snps2 snp3 snp4

**#AL513382.1_phage_regions.bed (available at https://phaster.ca/submissions/AL513382.1)**

AL513382 915628 944619

AL513382 1008698 1053060

AL513382 1538899 1572919

AL513382 1767007 1791034

AL513382 1883242 1936194

AL513382 1909350 1940164

AL513382 2461018 2465770

AL513382 3504242 3555052

AL513382 4462428 4509535

AL513382 4681892 4696511

**Genotyping**

#**Run GenoTyphi v1.9.1 using CT18**

python genotyphi.py --mode bam --bam *.bam --ref AL513382.fasta --ref_id AL513382.1 --output genotypes.txt

**Maximum-likelihood phylogeny and pairwise SNP distance calculation**

**#Run NASP v1.2.2**

format_fasta --inputfasta /input/STRAIN.fasta --outputfasta /results/STRAIN.fasta

convert_external_genome --nucmerpath nucmer --nucmerargs '' --deltafilterpath delta-filter --deltafilterargs '' --reference reference.fasta --external /results/STRAIN.fasta --name STRAIN

submit_command = while kill -0 846216; do sleep 300; done; while [ `free -m | grep cache: | awk '{ print $4 }'` -lt 93750 ]; do sleep 300; done; format_fasta --inputfasta /input/STRAIN.fasta --outputfasta /output/STRAIN.fasta; convert_external_genome --nucmerpath nucmer --nucmerargs '' --deltafilterpath delta-filter --deltafilterargs '' --reference reference.fasta --external /output/STRAIN.fasta --name STRAIN

##repeat for each strain##

nasptool_linux_64 matrix --dto-file matrix_dto.xml --num-threads 8

while [ -s nasp_matrix_dependent_pids ]; do sleep 600; for pid in `cat nasp_matrix_dependent_pids`; do kill -0 "$pid" 2>/dev/null || sed -i "/^$pid$/d" nasp_matrix_dependent_pids; done; done; rm nasp_matrix_dependent_pids; while [ `free -m | grep cache: | awk '{ print $4 }'` -lt 93750 ]; do sleep 300; done; nasptool_linux_64 matrix --dto-file matrix_dto.xml --num-threads 8

nasptool_linux_64 export --type vcf bestsnp.tsv > bestsnp.vcf & nasptool_linux_64 export --type vcf missingdata.tsv > missingdata.vcf & nasptool_linux_64 export --type fasta bestsnp.tsv > bestsnp.fasta & nasptool_linux_64 export --type fasta missingdata.tsv > missingdata.fasta & wait

while kill -0 854317; do sleep 300; done; while [ `free -m | grep cache: | awk '{ print $4 }'` -lt 93750 ]; do sleep 300; done; nasptool_linux_64 export --type vcf bestsnp.tsv > bestsnp.vcf & nasptool_linux_64 export --type vcf missingdata.tsv > missingdata.vcf & nasptool_linux_64 export --type fasta bestsnp.tsv > bestsnp.fasta & nasptool_linux_64 export --type fasta missingdata.tsv > missingdata.fasta & wait

**#Run Gubbins v2.4.1 with GTR-Gamma model**

run_gubbins.py bestsnp.fasta --raxml_model GTRGAMMA

**#Run RAxML v8.2.12 on PHYLIP format alignment of filtered polymorphic sites**

raxmlHPC-PTHREADS -f a -x 12345 -p 12345 -# 100 -m ASC_GTRGAMMA -s bestsnp.filtered_polymorphic_sites.phylip -n rax_tree -T 16 --asc-corr=lewis

**#Run snp-dists v0.8.2**

snp-dists bestsnp.fasta > bestsnp_snpdists.tsv

**Gene content comparison**

**#Run LS-BSR**

python ls_bsr.py -k T -s F -a 10 -i 0.9 --ml 0.9 -b blastn -d /genomes -c cd-hit-est

**#Convert LS-BSR matrix to Scoary matrix with cutoff = 0.9**

python BSR_to_scoary.py -l 0.9 -b bsr_matrix.txt

**#Run Scoary with Bonferroni adjust p-value of 0.05**

scoary -g Scoary_matrix.csv -t traits.csv -p 0.05 -c B

**Temporal analysis**

**#Follow steps for Snippy v4.6.0 and Snippy-core v4.6.0 (see above)**

**#Follow steps for Gubbins v2.4.1 and RAxML v8.2.12 (see above)**

**#Decimal_Dates in R, generic example**

library(lubridate)

random = read.table("ymd_input.txt")

random2 = ymd(random$V1)

final = decimal_date(random2)

write.table(final, file="decimal_date_output.txt")

**#TempEst v1.5.3**

#Load resulting NEWICK tree into TempEst

#Assign sampling times using decimal dates

#Perfrm regression analysis of root-to-tip branch distances as a function of sampling times

#Select best-fitting root and heuristical residual mean squared

**Evolutionary analysis**

**#BEAUTi v1.10.4 settings:**

1. PARTITIONS – drag and drop core_LT904890.1_ymd.fasta (renamed from core.aln output file from snippy-core, and taxa headers contain YYYY-MM-DD dates)

2. TAXA – no changes

3. TIPS – import decimal dates file. Parse as number. Manually add uncertainty

3. TIPS – parse Dates. Parse calendar dates with variable precision. Fix precision for any that need fixing, like the 1980s strains.

4. TRAITS – no changes

5. SITES

- Substitution Model: GTR

- Base frequencies: Empirical

- Site Heterogeneity Model: Gamma

- Number of Gamma Categories: 4

6. CLOCKS

- Clock Type: Uncorrelated relaxed clock

- Relaxed Distribution: Lognormal

7. TREES

- Tree Prior: Coalescence: Exponential Growth

8. STATES – no changes

9. PRIORS

- gtr.rates: Dirichlet [1,1]

- alpha: Exponential [0.5], initial=0.5

- ucld.mean: Approx. Reference Prior: CTMC Rate Reference

- ucld.stdev: Exponential [0.333333,], intial = 0.333333

- treeModel.rootHeight: Using Tree Prior in [##.####, inf]

- exponential.popSize: Exponential [1E5], initial=1

- exponential.growthRate: Laplace [0,1], initial=0

10. OPERATORS - no changes

11. MCMC

- Length of Chain: 200000000

- Echo...: 20000

- Log....: 20000

- Name file stem

- Marginal likelihood estimate (MLE): generalized stepping-stone sampling

- Settings > Tree working prior: product of exponential distributions

Number of stepping stones: 200

Length of chains: 1000000

Log likelihood every: 1000

**#Open XML file and replace patterns with**

<mergePatterns id="patterns">

<patterns from="1" every="1">

<alignment idref="alignment"/>

</patterns>

<constantPatterns>

<alignment idref="alignment"/>

<counts>

<parameter value="1067881 1159880 1163817 1070422"/> <!-- This is the number of A C G and T -->

</counts>

</constantPatterns>

</mergePatterns>

**#Generate three runs of BEAST xml for each reference, Samoa and CT18; example**

beast run1.xml

**#Summarize parameter estimates**

#The .log files were loaded into Tracer v1.7.1 to verify that all EES values were >200

**#Build the MCC tree using TreeAnnotator v1.10.4**

#Burn-in: 20,000,000 (10% of total)

#Target tree type: MCC

#Node heights: Median heights

#Input file is *.trees

#Output file is *.mcc.tre saved into an MCC subdirectory

**#Visualize MCC tree in R**

setwd("path/to/MCC")

library(ggtree)

library(treeio)

library(ggplot2)

library(RColorBrewer)

country <- read.csv("country.csv")

tree <- read.beast("beast.mcc.tre")

cols <- c(Chile='#d73027', Colombia='#f46d43', Indonesia='#fdae61', Vietnam='#fee090', Laos='#e0f3f8', Unknown='#abd9e9', Fiji='#74add1', Samoa='#4575b4')

ggtree(tree, right=TRUE, mrsd="2020-06-09") %<+% country +

geom_tippoint(aes(color=Country), size=1.5, alpha=0.75) +

scale_color_manual(values=cols) +

theme_tree2(legend.position='right') +

scale_x_continuous(breaks=seq(1870, 2020, 10), minor_breaks=seq(1870, 2020, 5)) +

xlab("Year") +

theme(panel.grid.major = element_line(color="black", size=.2),

panel.grid.minor = element_line(color="grey", size=.1),

legend.title=element_text(face="bold", size=14),

legend.text=element_text(size=14),

legend.key.height=unit(.6, "cm"),

legend.key.width=unit(1, "cm"),

legend.position=c(.18, y=0.4),

axis.text.x=element_text(color="black",face="bold",size=14),

axis.title.x=element_text(face="bold",size=14))
